# Quality of life in the COVID-19 outbreak: influence of psychological distress, government strategies, social distancing, and emotional recovery

**DOI:** 10.1016/j.heliyon.2021.e06407

**Published:** 2021-03-02

**Authors:** Abdul Gaffar Khan, Md. Kamruzzaman, Md. Nannur Rahman, Monowar Mahmood, Md. Aftab Uddin

**Affiliations:** a Department of Management, Mawlana Bhashani Science and Technology University, Tangail 1902, Bangladesh; b Dept. of Applied Nutrition and Food Technology, Islamic University, Kushtia 7003, Bangladesh; c Dept. of Food Technology and Nutritional Science, Mawlana Bhashani Science and Technology University, Tangail 1902, Bangladesh; d Independent Researcher, Chattogram, Bangladesh; e Department of Human Resource Management, University of Chittagong, Chattogram 4331, Bangladesh

**Keywords:** COVID-19, Social distancing, Government strategies, Psychological distress, Emotional recovery, Quality of life

## Abstract

Considering the severity of the effects of COVID-19 on psychological health and quality of life, the present study investigates the direct effects of government strategies and social distancing and the moderating effect of emotional recovery on psychological distress and quality of life using the tenets of the theory of attachment and learned helplessness. The snowball sampling technique was used to recruit respondents from Bangladesh who completed a self-administered questionnaire via Google Forms, which provided cross-sectional data. The results revealed that both social distancing and government strategies have significant negative influences on psychological distress. Besides, government strategies have a significant positive influence on social distancing. Although psychological distress has a significant negative influence on quality of life, emotional recovery shows no moderating effect on the relationship between psychological distress and quality of life during the COVID-19 pandemic. The study provides insights for regulatory bodies and policymakers for developing effective policy interventions to ensure the well-being of people during this pandemic. Finally, the study highlights the implications for both theory and practice and a few notes for further research.

## 1 Introduction

The recent 2019 outbreak has become a threat and put humankind into great trouble. SARS-COV-2, also known as COVID-19, was first identified in Wuhan, China's wet market (Maugeri et al., 2020; Shi et al., 2020a, Shi et al., 2020b). Due to the high reproduction coefficient (R_0_ = 3.84) (Liu et al., 2020), the SARS-COV-2 virus spread worldwide rapidly, and the incidence rate rises at an unprecedented speed. As of December 15, 2020, more than 73,452,304 individuals have been reported as infected worldwide (218 countries), and this number is 494,209 for Bangladesh (JHU, 2020; Worldometers, 2020).

The COVID-19 pandemic has created panic among the population, both infected and vulnerable people (Melo and Soares, 2020; Zubayer et al., 2020). Health authorities, however, in most countries, including Bangladesh, mainly focus on controlling the number of new cases and deaths to a minimum, but psychological distress and its associated reverberations are often overlooked. Psychological distress does not emerge only from the panic of being infected with COVID-19; several other factors also play rigorous roles (Islam et al., 2020; Mahmud et al., 2020; Nyarko et al., 2020).

Most countries follow social distancing as the principal strategy to limit the number of new cases (Das et al., 2020; Melo and Soares, 2020). The critical part of social distancing is creating loneliness, disrupting regular activities, limiting freedom of movement, decreasing employment and income, and creating a lack of medical services to treat diseases other than COVID-19 (Kaufman et al., 2020; Ripon et al., 2020; Serafini et al., 2021). Experience from countries such as China, Singapore, South Korea, and Japan endorsed that government strategies such as social distancing effectively contain the further spread and transmission of COVID-19 and lead to moderate psychological distress (Sajed and Amgain, 2020).

Psychological distress, both long and short term, may interfere with quality of life unless proper preventive measures are taken for rampant emotional recovery (Panthee et al., 2020; Shechter et al., 2020). To grasp these objectives and justify our research model during the COVID-19 outbreak, the present study proposes addressing the following research questions throughout the study:

**RQ1.** How do social distancing and government strategies influence psychological distress?

**RQ2.** Does psychological distress impact quality of life?

**RQ3.** Is there any moderating effect of emotional recovery on the influence of psychological distress on quality of life?

## 2 Theory and hypotheses development

### 2.1 Social distancing

Social distancing decreases the interactions between individuals in a broader community so that the infection of any disease can be contained (Wilder-Smith and Freedman, 2020). Thus, people will have less chance of being exposed to the virus and being infected. Even though this distancing makes people feel lonely (Fawaz and Samaha, 2020; Mukhtar, 2020), considering the disastrous consequences of this infectious disease, they are happy that they will not suffer (Melo and Soares, 2020). In contrast, Wiederhold (2020) found that individuals during the COVID-19 outbreak might experience extreme psychological cessation, i.e., loneliness, boredom, and anger, due to the social distancing, quarantine, and isolation (Cénat et al., 2021; Shacham et al., 2020; Zubayer et al., 2020). In line with attachment theory's conceptualization, we posit that social distancing contributes to increased psychological distress due to attachment insecurity and insecurity during the COVID-19 pandemic (Byrne et al., 2017). Thus, we hypothesized the following:

H1 Social distancing has a significant influence on psychological distress.

### 2.2 Government strategies

Government strategies include the formulation of strategic and disciplinary initiatives to confine the spread of COVID-19. To fight the COVID-19 pandemic outbreak, Duan et al. (2020) revealed that the government's prevention control and rescue strategies significantly increased the public adoption of protective actions. Accordingly, governments can craft quick response strategies, such as lockdowns, social distancing, self-isolation, and home and institutional quarantines, by employing security forces and nongovernment organizations to deliver relief funds and medical facilities. During outbreaks and natural crises, people expect support from governments or nongovernment organizations to ensure their peace and happiness. Otherwise, insufficient government aid will foster psychological distress and anxiety among affected people and their surroundings. The government also arranges awareness programs and deployed forces to ensure self-isolation and social distancing (Siddika and Islam, 2020). Moreover, the government controls mass movements and crowds by shutting down shopping malls, educational institutes, public transports, sports, cinemas, parks, and tourist spots, directly influencing social distancing. Hence, we form the following hypotheses.

H2 Government strategies have a significant influence on psychological distress.

H3 Government strategies have a significant influence on social distancing.

### 2.3 Psychological distress

Psychological distress refers to unpleasant feelings or emotions and psychological discomfort that adversely affect the normal functioning of human beings (Rey et al., 2016). Studies evidenced that psychological distress influences the quality of life (Asadi et al., 2019; Gamage et al., 2020; Ramirez et al., 2012). Gamage et al. (2020) found that existential anxiety significantly diminished patients' quality of life. The COVID-19 outbreak creates more panic and illness and negatively influences quality of life (Uysal et al., 2016).

Conversely, lower psychological distress develops a commitment of emotions and self-efficacy, which, in turn, improves quality of life (Cummins, 2005; Shacham et al., 2020). Zhang and Ma (2020) reported that the COVID-19 outbreak caused mild psychological stress, i.e., physical and mental health, and this psychological stress influenced quality of life. Furthermore, learned helplessness theory states that the helplessness of individuals with psychological disorders may result in a low quality of life (Abramson et al., 1978; Teitelman and Priddy, 1988). Consequently, psychological stress has a powerful negative effect on quality of life (Abramson et al., 1978). Accordingly, we hypothesize as follows:

H4 Psychological distress has a significant influence on quality of life.

### 2.4 The moderating role of emotional recovery

Emotional recovery is an individual's personal trait that assists in releasing negative vibes or emotions (Akram et al., 2019; Li and Ahlstrom, 2016). Notably, the negative psychological consequences buffer strong personality traits, such as emotional recovery ability (Li and Ahlstrom, 2016; Panthee et al., 2020). Persons with low emotional recovery capability usually experience psychological outbreaks (Ramirez et al., 2012). Therefore, emotional recovery moderates the negative feelings and stress from unexpected situations (Li and Ahlstrom, 2016). People with higher emotional recovery do not endure depression or somatization of psychological distress, enhancing their quality of life, and vice versa (Li and Ahlstrom, 2016; Ramirez et al., 2012). Consequently, it seems that during the COVID-19 pandemic, psychological distress affecting quality of life will disappear when emotional recovery occurs. In contrast, emotional recovery lessens the intensity of psychological distress, which consequently activates psychological peace or soundness. Thus, emotional recovery moderates the direct influence of psychological distress on quality of life in such a way that this association is weakened when emotional recovery is high, and vice versa. Thus, we state the following hypothesis.

H5 Emotional recovery moderates the negative relationship between psychological distress and quality of life such that the negative association is weaker (stronger) when emotional recovery is high (low).

**Figure 1:**
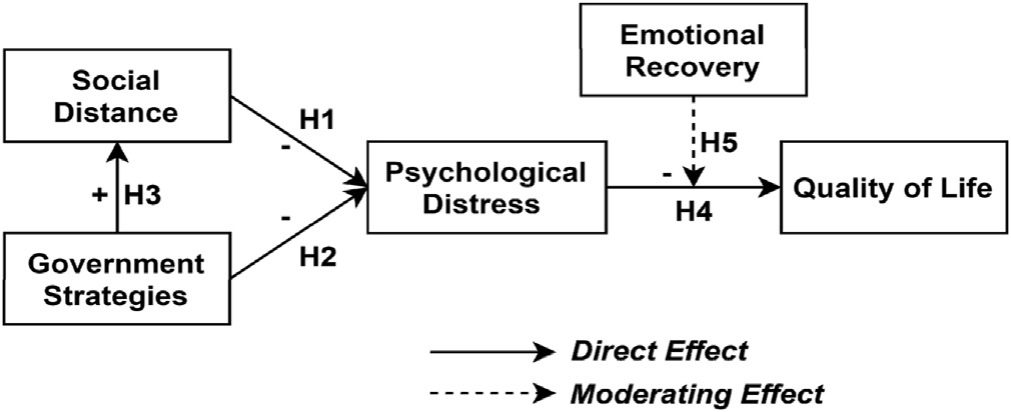
Proposed research model.

The hypothesized relationships in Figure 1 demonstrate the research model highlighting the influences of social distancing and government strategies on psychological distress. Subsequently, the figure depicts the consequential impact of psychological distress on quality of life. Finally, the research model displays the moderating influence of emotional recovery on the influence of psychological distress on quality of life.

## 3 Methodology

### 3.1 Study design

We opted to use an online survey to collect data from different social groups of Bangladesh, such as teachers, government officials, development workers, professionals, and students, through the snowball sampling technique because social distancing is a crucial issue during this COVID-19 outbreak (Roy et al., 2020; Shi et al., 2020a, Shi et al., 2020b). The ability to use an online survey is attributed to the proliferation of internet usage in Bangladesh due to the heightened government initiatives for digitalization (Shammi et al., 2020). The survey questionnaire has three sections. Section 1 includes a brief explanation, the aims, and statements regarding the confidentiality and anonymity of the study; section 2 measures the demographic characteristics of the respondents; and section 3 includes items to measure the latent variables. To avoid social desirability bias and response bias, we guaranteed that all data will be used for academic and research purposes by ensuring the respondents' privacy and anonymity (Maugeri et al., 2020). Additionally, the respondents were reminded that their participation is voluntary and can be withdrawn at any time during the data collection process. The online survey was administered using Google Forms and posted on social media, such as Facebook, LinkedIn, WhatsApp, etc.

Respondents aged 18 years and above with access to the internet were asked to respond to this survey. The uploaded survey materials were circulated for ten days using snowballing techniques. The determination of the sample size is a crucial issue for applying structural equation modeling (SEM). Although the Partial Least Squares-SEM (PLS-SEM) is flexible enough to generate results even from fewer than 100 samples, the existing literature attests that the minimum and optimum ratio must be from 5:1 to 10:1 (responses per item in the scale) to increase confidence in the results (Goodhue et al., 2006). Kline (2011) advocated using a minimum of 200 samples when applying SEM. Eventually, we received 481 responses and retained 465 responses after eliminating surveys with missing responses, outliers, and incomplete cases or responses (Fan et al., 2019; Mahmood et al., 2019). Thus, the sample size in this study is sufficient to generate the desired results.

### 3.2 Materials and instruments

To develop measurement tools, we adopted the back translation method documented in prior studies (mentioned in Appendix 1) and applied it. We scrutinized the expert opinions of a panel of researchers, health professionals, academics, and potential respondents to make few changes to the items to adapt them to Bangladesh perspectives (Brislin, 1970). We used a five-point Likert scale ranging from 5 (strongly agree) to 1 (strongly disagree) to score the items. Psychological distress is measured in three dimensions, including depression, anxiety, and somatization. The other parameters, such as psychological distress, emotional recovery, and quality of life, are measured following Meijer et al. (2011), Li and Ahlstrom (2016), and Whoqol (1995), respectively. Despite there are many scales to measure psychological distress, the study used the scale of Meijer et al. (2011) because this measurement scale is widely used in psychology and medical research (Baldassarri et al., 2020; Pachankis et al., 2020). Unlike the scales of Calderon et al. (2020) and Mewton et al., (2016), this scale provides robustness and complete cognitive reactions of psychological distress with the inclusion of anxiety, depression, and somatization. Additionally, during our pilot study, the scale of Meijer et al. (2011) was considered good fit considering respondents and Bangladeshi context.

Since COVID-19 is a very current issue, measurement tools for social distancing behavior and government mitigation strategies have not yet been developed. Thus, we developed a scale for social distancing based on previous studies (Kirk and Haegele, 2019; McBride et al., 2020). Likewise, the scale for ‘government strategies’ to contain the COVID-19 outbreak is measured using six items based on Ilyas et al. (2020) and Siddika and Islam (2020).

Following the procedures mentioned in Hasan and Bao (2020), Azim et al. (2020) and Miah et al. (2017), the present study adopted the mixed method-quantitative approach by conducting a pilot study and –qualitative method through an online focus group study among a group of potential respondents from diverse backgrounds, including different genders, educations, ages, incomes, geographical locations, etc. Finally, independent t-tests were run among the first 50 and last 50 responses to observe the stability of the construct at two different times (T1 and T2) and the nonresponse bias (Armstrong and Overton, 1977), and we observed no significant variance in T1 and T2 (Azim et al., 2019; Azim et al., 2020) with a significant correlation (p < 0.01) between T1 and T2 (Ding and Yu, 2020).

The researchers conducted a pilot study among 15 health professionals and 15 academics to explore issues with the understanding and operationalization of the scale, and minor modifications were made based on the feedback to ensure the ease comprehension of the items (Lins and Aquino, 2020; Shammi et al., 2020). The estimated Cronbach's alpha and composite reliability for government strategies and social distancing were 0.857 and 0.896 and 0.925 and 0.947, respectively, which are within the threshold limit (Hair et al., 2014, 2017). Besides, the average variances extracted (AVEs) of both scales (government strategies = 0.593 and social distancing = 0.816) are also within the cutoff value (Hasan and Bao, 2020; Zhang, 2007).

### 3.3 Statistical analysis

We refined the datasets using Microsoft Excel and screened data and generated estimates by running Smart PLS-SEM (SmartPLS-version 3) to examine the proposed hypotheses. SmartPLS-led structural equation modeling is a second-generation regression technique that uses a measurement model to assess the psychometric properties of scales (Lins and Aquino, 2020) and a structural model to assess the hypothesized relationships (Hair et al., 2017). Both measurement and structural models are indispensable parts of SEM. They are tested by examining the reliabilities and validities to confirm the psychometric properties of scales and assess the path coefficients, significance levels, and p-values to assess the observed relationships (Mahmood et al., 2020).

### 3.4 Compliance with ethical standards

This research was administered with ethical approval [reference no: mbstu/b.ad/others/15/327/2020(3)] from the ethical review committee of the Department of Business Administration, Faculty of Business Studies, Mawlana Bhashani Science and Technology University, Bangladesh. The study was conducted following the 1964 Declaration of Helsinki and its later amendments. The aims, potential risks, benefits, and confidentiality were made known to the respondents in an informed consent form. Additionally, participation was voluntary, and we gave them the full right to refuse or accept to respond. The anonymity and confidentiality of the data were assured.

## 4 Results

### 4.1 Participants

The majority of the respondents were male (71 percent). The percentage of unmarried respondents (63 percent) was greater than the percentage of married respondents. The most significant number of respondents were 31 years–40 years old because youths have more internet access than old adults in Bangladesh (Shammi et al., 2020). A total of 218 respondents completed master's degrees, 167 completed undergraduate degrees, 64 completed below an undergrad education, and 16 completed PhDs. We observed that 44 percent of respondents came from middle-income groups (205), and the remaining respondents were from lower-income groups (33 percent) and higher-income groups (23 percent).

### 4.2 Descriptive analysis

The descriptive statistics are illustrated in Table 1 through the disclosure of the mean and standard deviation of variables. Additionally, Table 1 also shows the estimated correlations among variables. The estimated correlations revealed that all latent variables were significantly associated (p < 0.01).

**Table 1:** Estimates on reliabilities and validities.

Variables	1	2	3	4	5	6	7	8	9	10	11	12
**Control variables**												
1. Age	1											
2. Gender	-.253∗∗	1										
3. Education	.697∗∗	-.285∗∗	1									
4. Marital status	-.640∗∗	.131∗∗	-.520∗∗	1								
5. Income	.457∗∗	.002	.344∗∗	-.444∗∗	1							

**Latent variables**												
6. ER	-.005	-.018	.002	.054	-.032	***0.866***						
7. GS	-.086	.000	-.106∗	.112∗	-.130∗∗	0.236∗∗	***0.770***					
8. PsA	-.030	-.055	-.009	-.016	.033	-0.235∗∗	-0.283∗∗	***0.871***				
9. PsD	.074	-.053	.063	-.068	.105∗	-0.244∗∗	-0.377∗∗	0.577∗∗	***0.870***			
10. PsS	-.019	-.084	.041	-.025	.011	-0.277∗∗	-0.285∗∗	0.548∗∗	0.591∗∗	***0.904***		
11. QL	.006	.040	.008	.034	-.052	0.393∗∗	0.158∗∗	-0.467∗∗	-0.448∗∗	-0.480∗∗	***0.773***	
12. SD	-.030	.102∗	-.048	.042	-.067	0.265∗∗	0.369∗∗	-0.284∗∗	-0.468∗∗	-0.167∗∗	0.294∗∗	***0.903***
Cronbach's Alpha						0.917	0.857	0.920	0.920	0.944	0.904	0.925
Composite Reliability						0.938	0.896	0.940	0.940	0.957	0.922	0.947
Average Variance Extracted (AVE)						0.751	0.593	0.759	0.757	0.817	0.598	0.816
Mean						3.779	3.860	2.061	2.119	2.405	3.738	4.248
Std. Deviation						.768	.698	.834	.941	1.078	.747	.869

ER. Emotional recovery, GS. Government strategies, PsA. Psychological anxiety, PsD. Psychological depression, PsS. Psychological somatization, QL. Quality of life, SD. Social distancing.

∗∗ Correlation is significant at the 0.01 level (2-tailed), ∗ Correlation is significant at the 0.05 level (2-tailed).

### 4.3 Measurement model evaluation

We assessed the measurement model using the convergent validity and discriminant validity. Table 1 highlights that the minimum reliability of the latent variable (Cronbach's alpha = 0.857 and composite reliability = 0.896) is above 0.80, and the lowest AVE of any latent variable is 0.593 (government strategies), which is also above the threshold limit of 0.50 (Hair et al., 2017). One item each was dropped from psychological depression, psychological anxiety, and psychological somatization because of their low regression weights. Discriminant validity was tested using the square root of the AVE. Table 1 also depicts that the square root of a latent variable's AVE is higher than its correlation with other latent variables, which confirms the discriminant validity. Thus, both discriminant validity and convergent validity are supported.

### 4.4 Hypothesis testing in structural model

**Figure 2:**
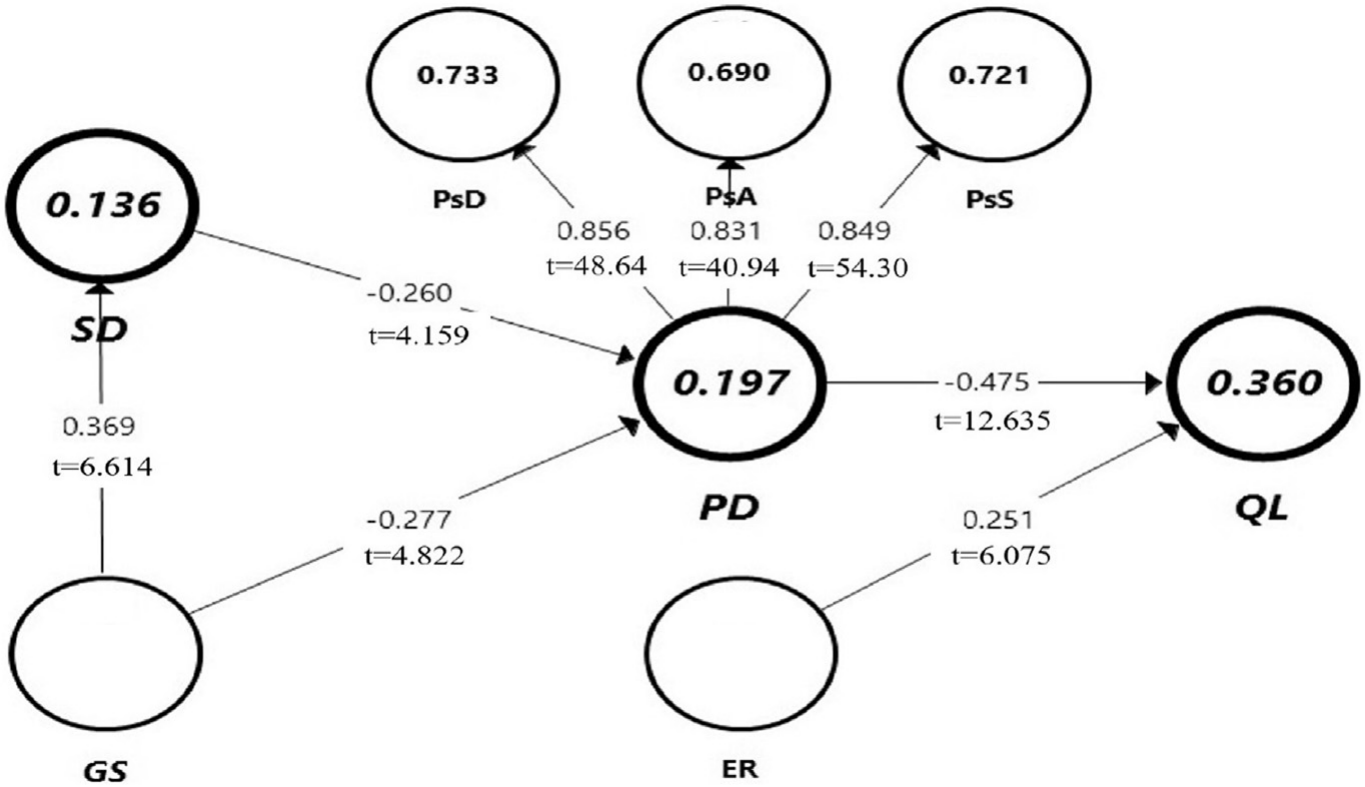
Structural model. GS. Government strategies, SD. Social distancing, PD. Psychological distress, QL. Quality of life, ER. Emotional recovery.

We estimated the structural model using 5000 bootstrapping cases to examine the hypotheses (Hair et al., 2017). We examined the βs and p-values and found that they are satisfactory because the paths are significant at p < 0.000. Figure 2 displays the estimates of the underlying direct effects of the independent variables on the dependent variables. In hypothesis 1, we predicted that social distancing influences psychological distress. The results revealed that the influence is negative and significant (β = -0.260, p = 0.000). Therefore, H1 is supported. We hypothesize in H2 that government strategies have a significant influence on psychological distress. As desired, we found that the effect is negative and significant (β = -0.277, p = 0.000). Thus, H2 is supported. We proposed in hypothesis 3 that government strategies influence social distancing. The results showed that the influence was positive and significant (β = 0.369, p = 0.000). Hence, H3 is supported. Finally, in hypothesis 4, we hypothesized that psychological distress significantly influences quality of life. The estimates in Figure 2 support that psychological distress negatively affects quality of life, and the influence is significant (β = -0.475, p = 0.000). Thus, H4 is also supported.

### 4.5 Examining the moderating effects

We hypothesized that emotional recovery moderates the influence of psychological distress on quality of life. Using SmartPLS 3, we also examined the moderating influence of the moderator variable. Table 2 demonstrates that the direct effects from both psychological distress to quality of life (β = -0.475, p = 0.000) and from emotional recovery to quality of life (β = 0.249, p = 0.000) are significant; however, the moderating effect is not significant (β = 0.008, p = 0.804). Thus, there is no moderating influence of emotional recovery on the influence of psychological distress on quality of life. Thus, hypothesis 5 is also not supported.

**Table 2:** Moderating effect of emotional recovery.

Path relations	Β	Standard Error	T Statistics	P Values	Decision
PD → QL	-0.474	0.042	11.241	0.000	Not supported
ER → QL	0.249	0.040	6.161	0.000	
ER∗PD→QL	0.008	0.031	0.248	0.804	

PD. Psychological distress, QL. Quality of life, ER. Emotional recovery.

## 5 Discussion and conclusion

This study investigated the relationships of government strategies and social distancing on psychological distress, which affects quality of life. The study revealed that (1) social distancing is negatively related to psychological distress, (2) government strategies negatively contribute to psychological distress, (3) government strategies have a positive influence on social distancing, (4) psychological distress negatively influences quality of life, and (5) the insignificant negative relationship between psychological distress and quality of life is reduced when emotional recovery is sufficiently high.

### 5.1 Implications for theory

Our study contributes to the theory by extending the psychological consequences of the COVID-19 outbreak and improving quality of life. First, we contribute to unmasking the regulators of psychological distress from COVID-19 outbreaks by assessing the direct effects of preventive health measures, i.e., social distancing and government strategies, on psychological distress. Prior studies advocated that an epidemic's adversities worsen psychological health (Coroiu et al., 2020; Qiu et al., 2020; Wang et al., 2020).

The findings in this study are also not unlikely because the outbreak of COVID-19 magnifies psychological distress. However, governments ensuring social distancing and implementing appropriate containment strategies significantly reduce or eliminate psychological distress by limiting community transmission among the masses. Thus, the outcomes of our study enrich the literature on preventive health measures during the spread of COVID-19. Second, our findings extend the literature on quality of life by exploring the significant negative influence of psychological distress on quality of life during this abnormal time. The findings postulate that subjective wellbeing among people during COVID-19 is worse due to increased uncertainty and anxiety regarding COVID-19 (Satici et al., 2020; Shechter et al., 2020).

Surprisingly, the results show that there is no significant moderating effect of emotional recovery on the relationship between psychological distress and quality of life. Using the lens of learned helplessness theory, the study posits that this occurs because people are too distressed by COVID-19 phobia to lower their tension due to any supplements, i.e., emotional recovery. Thus, the findings of this moderating effect of emotional recovery postulate that policymakers might focus more on quick and permanent solutions to improve the quality of life during a health crisis rather than augmenting internal intent (Sahoo et al., 2020).

Finally, the study's results are novel in that they connect the proposed model with attachment theory and learned helplessness theory during a panic situation. In a way, the observed associations are consistent with the tenets of these two theories. It provides evidence for the inclusion of a multitheory perspective to integrate these fragmented concepts comprehensively. This study's observed results also help understand the outbreak thoroughly, which could help design further research for upcoming diseases or outbreaks.

### 5.2 Implications for policy interventions

Our findings may apply to contain the current COVID-19 outbreak or future outbreaks by government bodies and the general population by integrating policy interventions. First, the first hypothesis confirms a significant and negative relationship between social distancing and psychological distress. More precisely, people have regarded social distancing as a positive phenomenon that reduces psychological distress (Bjursell, 2020). One possible explanation of our negative correlation could be that most of the participants were male (71%, Table 2), and Lai et al. (2020) reported that females are more likely to suffer from severe depression due to the COVID-19 outbreak.

The majority of our participants were educated, particularly at the level of undergraduate education and over; they had a good income level (>30,00 BDT, Table 2) and they were young (18–40 years, Table 2). Young and educated persons tend to find COVID-19-related information from social, print, and electronic media (Qiu et al., 2020), which causes them to prioritize not becoming infected. In addition, during the first stage of the outbreak and lockdowns, people were less likely to experience an economic crisis, which might have negative psychological consequences in the long run.

Furthermore, our third hypothesis reveals that government strategies have positive and significant influences on social distancing, which supports the link of the second hypothesis with the first and third hypotheses. The second hypothesis is that government strategies significantly influence psychological distress, which is also supported by our statistical outcome. Thus, the present study opens a window of opportunity for the government to consider short- and long-term lockdowns as strategic planning, a preventive measure of COVID-19, and the consequences.

The study found that psychological distress and emotional recovery have direct influences on quality of life. However, the moderating influence of emotional recovery on quality of life was not significant. Our finding agreed with the findings of Lau et al. (2006) and Zhang and Ma (2020). Thus, from the current results, it is comprehensible that emotional recovery could be a viable option to support people on the verge of psychological distress. Few previous studies also support this phenomenon (Saltzman et al., 2020; Zhang et al., 2020). The present study may be instrumental in preparing the community and the country at large so that an engaging and effective strategic focus can be instilled and upgraded in the future to prevent a catastrophe such as COVID.

### 5.3 Limitation and future research directions

Despite its remarkable prospects, this study has few limitations. First, this study selected small samples for the research, and the majority of respondents were male, which makes it difficult to generalize the study results. Second, the study has been conducted in Bangladesh only, which results in cross-nation limitations. Therefore, a longitudinal study could be undertaken with larger samples representing equal portions of males and females throughout the country to improve the generalization of the findings.

Future research may study different COVID-19 patients and nonpatients in a multicountry context because economic conditions, health systems, immune systems, physical conditions, cultural values, and weather conditions differ from one country to another. Finally, our study is nonexperimental research that will prevent us from drawing inferences on pandemic outbreaks such as COVID-19. Meanwhile, we did not conduct multiple time-lagged surveys, which is another limitation. This limitation may create an ambiguous causality problem. Future researchers might conduct experimental research in both the laboratory and field with multiple wave survey data to capture accurate causality among these variables.

## Declarations

### Author contribution statement

Md. Aftab Uddin, Abdul Gaffar Khan, Md. Nannur Rahman, Monowar Mahmood, Md. Kamruzzaman: Conceived and designed the experiments; Performed the experiments; Analyzed and interpreted the data; Contributed reagents, materials, analysis tools or data; Wrote the paper.

### Funding statement

This research did not receive any specific grant from funding agencies in the public, commercial, or not-for-profit sectors.

### Data availability statement

Data will be made available on request.

### Declaration of interests statement

The authors declare no conflict of interest.

### Additional information

No additional information is available for this paper.

## Figures and Tables

**Table:** 

Measures	Items
Social distancing	Nowadays, I maintain physical distance with outside people in society.
	Nowadays, I retain physical distance from my colleagues with whom I work.
	Nowadays, I keep on physical distance with my friends and relatives that I meet often.
	Nowadays, I maintain physical distance with my family members when I need to work outside.
Government strategies	Government set policy framework on time for incorporating social, health and security measures in fighting COVID-19
	The government started an immediate lockdown strategy for COVID-19 pandemic urging people to stay at home
	The government deployed various security forces for ensuring social distancing and self-isolation as a method of limiting the outbreak of COVID-19
	Government is also allowing initiative by NGO and private organizations to fight the outbreak of novel coronavirus
	Government is providing relief and medicare to the effected people on time
	Centralizing the testing facilities of coronavirus is increasing the spread of virus
Psychological distress-depression	I am feeling sad, dirty or unwell
	I am feeling no interest to do anything
	I am feeling lonely
	I am feeling hopeless
	I am feeling valueless/unemployed
	I am feeling suicidal thoughts
Psychological distress-Anxiety	I am really feeling tensed
	I am feeling nervous
	I am feeling fearful
	I am feeling spells of panic
	Suddenly, I am feeling scared without reason
	I am feeling restless
Psychological distress-somatization	I am feeling weak
	I am feeling vomit
	I am feeling numbness, emotionlessness
	I am feeling dullness and exhaustive
	I am feeling trouble for getting breath and respiration
	I am feeling chest pain
Emotional recovery	I can recover from unhappiness quickly and not be influenced by it
	I can calm down quickly from anxiety and not be influenced by it
	I can recover from upset quickly and not be influenced by it
	I can recover from panic (or scare/fear) quickly
	I can recover from negative emotions quickly
	I can recover from distress (or worry) quickly
Quality of life	The opportunity of education is going smooth
	Income and consumption is remaining same
	Productivity and creativity have increased due to lacking of paid and unpaid work
	The life expectancy and number of healthy life years remain same
	Comorbidities will be reduced
	Attending leisure activities including outdoor sports and cultural program increased
	Fulfilment of basic rights is not questionable
	Child and maternal health is still insured
